# In-vivo assessment of meniscal movement in the knee joint during internal and external rotation under load

**DOI:** 10.1186/s40634-022-00540-5

**Published:** 2022-10-04

**Authors:** Andreas Fuchs, Joachim Georgii, Elham Taghizadeh, Stefan Heldmann, Thomas Lange, Sebastian F. Bendak, Markus Siegel, Tayfun Yilmaz, Hagen Schmal, Kaywan Izadpanah

**Affiliations:** 1 Department of Orthopedic Surgery and Traumatology, Freiburg University Hospital, Albert Ludwigs University Freiburg, Hugstetter Straße 55, 79106 Freiburg, Germany; 2 Fraunhofer Institute for Digital Medicine MEVIS, Max-von-Laue Str.2, 28359 Bremen, Germany; 3 Department of Radiology, Medical Physics, Medical Center, Faculty of Medicine, University of Freiburg, University of Freiburg, Killianstrasse 5a, 79106 Freiburg, Germany; 4 Department of Orthopedic Surgery, University Hospital Odense, Sdr. Boulevard 29, 5000 Odense C, Denmark

**Keywords:** Meniscal Movement, In-vivo MRI, Internal and External Rotation, Dynamic MRI Evaluation

## Abstract

**Purpose:**

The menisci transmit load between femur and tibia and thus play a crucial role in the functionality of the knee joint. Knee joint movements have a major impact on the position of the menisci. However, these meniscus movements have not yet been assessed in a validated setting. The objective of this study is to evaluate the meniscal movements in MRI with prospective motion correction based on optical tracking under loading via internal and external tibial torques.

**Methods:**

Thirty-one healthy volunteers were recruited for this study. MRI scans were performed in internal and external rotation induced by a torque of 5 Nm, using a 3 T MRI. A validated software used the generated images to calculate the absolute meniscus movements as the sum of all vectors. Differences between subgroups were analyzed by using a Wilcoxon signed-rank test.

**Results:**

The MM shows an average movement of 1.79 mm in anterior-lateral direction under internal rotation and 6.01 mm in posterior-lateral direction under external rotation, whereas the LM moves an average of 4.55 mm in posterior-medial direction under internal rotation and 3.58 mm in anterior-medial direction under external rotation. When comparing the overall meniscus movements between internal and external rotation, statistically significant differences were found for total vector length and the direction of meniscus movements for medial and lateral meniscus. The comparison between medial and lateral meniscus movements also showed statistically significant differences in all categories for internal and external rotation.

**Conclusions:**

Overall, the MM and LM movements in internal and external rotation differ significantly in extent and direction, although MM and LM movements in opposite directions during internal and external rotation can be observed. In internal rotation, most meniscus movements were found in the IHLM. In external rotation, the IHMM showed the greatest mobility. Segment analysis of internal vs. external rotation showed less difference in LM movements than MM.

**Level of evidence:**

Level II.

## Background

The menisci transmit load between femur and tibia, and thus play a crucial role in the functionality of the knee joint. They enlarge the interaction between the incongruent femur and tibia in both compartments, thereby distributing forces and enhancing stability during the movement of the joint [6, 26]. They also contribute to shock absorption, nutrient distribution, joint lubrication, and joint stability [15]. An intact and functioning meniscus is crucial to prevent the onset and progression of osteoarthritis [18].

Knee joint movements have a major impact on the position of the menisci. Several studies have been conducted to investigate these meniscus movements, both in vivo and in vitro. Based on cadaveric studies, there is evidence that the menisci are mobile and allow movements in an anterior–posterior (AP) direction and in a medial–lateral (ML) direction [3, 22]. In vitro studies showed that increasing flexion leads to posterior movements in both menisci, medial and lateral [3]. Internal and external rotation moves both menisci in opposing directions on the tibial plateau. During all these motions, the lateral meniscus shows a greater range of movement on the tibial surface [3, 22, 24]. What all these studies have in common, however, is that they were carried out in non-validated and hardly reproducible settings.

As a diagnostic tool, MRI has gained importance for investigating cartilage and menisci over the last two decades as it provides high spatial resolution and high soft-tissue contrast. However, one of its main disadvantages is its limited ability to capture dynamic processes, due to its susceptibility to motion artifacts. Although in-vivo studies have tried to gain more information on the behavior of the meniscus with the help of MR imaging, the poor image quality of open-bore MRI and short sequence imaging to avoid motion artifacts have repeatedly led to a limited assessment of meniscus movements in in-vivo studies [2, 25]. Therefore, the experience with in-vivo imaging of the dynamic properties of the meniscus is limited for in vivo MR imaging of the knee under weight-bearing conditions [10, 11, 25].

One approach to visualizing the displacement of the menisci during joint loading is to attach radiographic markers on the menisci [3]. MRI has been used for the noninvasive evaluation of healthy and pathological meniscal tissue [21] and assessment of knee kinematics [17, 19] during weight-bearing. Other MRI studies have investigated meniscal movement during flexion [2, 10, 16, 19, 22, 23, 25, 29]. While these studies have improved our knowledge of the basic kinematic behavior of soft tissues, different limitations with respect to image evaluation and process validation limit the general informative value regarding clinical practice. However, the above-mentioned limitations can now be overcome with MRI augmented with prospective motion correction (PMC) and an MRI-compatible pneumatic device, to improve image quality and standardize knee positioning while staying inside a closed bore. Knee cartilage MRI with prospective motion correction based on optical tracking was proposed, and has been applied in a pilot study for investigating the patellofemoral cartilage contact and compression behavior under loading [13, 30].

The effect of internal and external tibial torque on the motion of the meniscus has not been investigated thoroughly. To gain these insights from biomechanical behavior in-vivo, it is crucial to evaluate the meniscus dynamics under load and in stress position. The exact determination of physiological movements in the different meniscus segments depending on torsional movements of the knee was the objective of this study. This could be of particular importance in the detection of specific meniscus injuries (e.g. meniscal ramp lesions). A validated in vivo examination method has not yet been carried out in this context, and could become a decisive addition to the preoperative diagnostics of knee injuries in the future.

## Methods

For this study, a population of 31 healthy volunteers was recruited. The mean age of the study population was 25.6 ± 4.4 (range, 21–42). The mean body mass index (BMI) was 22.7 ± 1.9 kg/m^2^ (range, 19.5 – 25.9), with a mean height of 176.8 ± 9.1 cm (range, 160—195) and mean weight of 71.2 ± 10.2 kg (range, 50 – 93). 15 (48.4%) study participants were females and 16 (51.6%) males. 13 (41.9%) right knees and 18 (58.1%) left knees were included in the present study. None of the volunteers had experienced any history of surgery on the knee joint, chronic pain or relevant trauma. Further exclusion criteria were any signs of degenerative changes or lesions on bone, cartilage, meniscal tissue and ligaments.

The study was approved by the institutional review board of the University Hospital Freiburg (Nr. 91/19 – 210,696) and the volunteers gave written informed consent prior to participation.

**Fig. 1:**
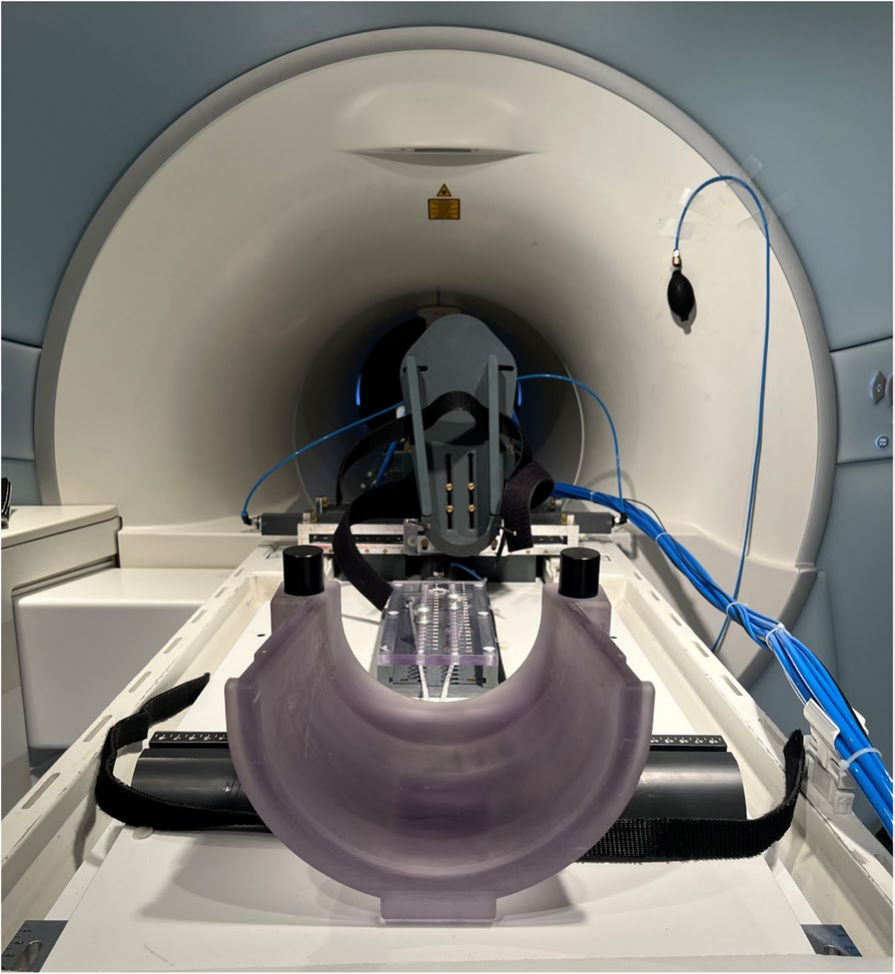
MR-compatible pneumatic device for positioning of the knee joint in internal and external rotation. * Below: femoral fixation with adjusted limitation to achieve a standardized positioning and knee flexion of 20°. ** Center: foot holder for applying internal and external rotation with a torque of 5 Nm and a constant axial load of 5 N

MRI scans were performed using a Magnetom Trio 3 T system (Siemens Healthineers, Germany) with an 8-channel multipurpose coil (NORAS MRI products, Germany). The examined leg was placed in an MR-compatible pneumatic device, which allowed the positioning of the knee joint in internal and external rotation (Fig. 1).

All MRI scans were performed with a T1-weighted spoiled 3D gradient-echo sequence using slab-selective water excitation and covering an FOV of 145 mm (AP) × 125 mm (RL) × 160 mm (FH) with a spatial resolution of 0.6 mm (AP) × 0.6 mm (RL) × 0.5 mm (FH), amounting to a scan time of 5:34 min. Further sequence parameters were: TR = 16 ms, TE = 6.88 ms, excitation angle = 15°, readout bandwidth = 130 Hz/Px, readout direction = FH. To mitigate motion artifacts, the sequence was augmented with PMC using a moiré phase tracking (MPT) system (Metria Innovation Inc., Milwaukee, US) [14]. For the accurate tracking of knee motion in all six degrees of freedom, this system consisted of a single in-bore camera and a single tracking marker, creating angle-dependent moiré patterns. Rigid-body motion was tracked with a frame rate of 80 frames per second, which enabled a position update of the MRI measurement volume before every excitation pulse.

Subjects were positioned on the device, then adjustments depending on height and proportions were made for each individual while correct foot and femoral fixation were ensured. Knee flexion was adjusted to approximately 20°, and the PMC tracking marker was attached to the center of the knee cap. The loading device was pneumatically controlled from the MRI console room. Knee flexion was achieved by elevating the thigh with the femoral fixation until the adjusted limitation of ~ 20°. A flexion angle of 20° was chosen because, due to the anatomical shape of distal femur and proximal tibia, no torsional movements in the knee joint are possible in full extension. 20° flexion turned out to be the joint position that allows torsional movements in the knee without relevant impairments but comes closest to extension and thus ensures the best possible reproducibility of the test setup. With the subject in place, scans were taken in internal and external rotation, with a torque of 5 Nm applied via the foot holder and a constant axial load of 5 N. Full imaging of each subject took approximately 45 min.

Segmentation of the femoral and tibial bone and cartilages, as well as of the lateral and medial menisci, were performed on the MR images. A combination of manual segmentation, deep learning and manual correction was used. From the manual segmentation with few samples, segmentation models for the bone, cartilage and menisci were trained. These models then were applied to pre-segment the rest of the images in the dataset, that were then corrected by clinical experts and included in the training set for the respective models.

To analyze meniscal displacement between different positions, an image-based registration pipeline was developed. First, images were aligned based on the tibial bone mask and a rigid registration was performed where the similarity measurement was restricted to the tibial volume. This approach provided all the images with a consistent coordinate system to analyze motions relative to the tibia.

The menisci were next registered nonlinearly. Here, a common variational approach [5] based on the curvature-based regularization of the computed deformation vector field and similarity measure of sum-of-squared-differences (SSD) was used, which evaluates the alignment of the bone masks. One problem with this approach is that it first requires a reasonable initial volume overlap. To overcome this problem, another penalty term that measures point-wise the distance between the meniscus faces was added, which can be efficiently computed by a distance transformation of the meniscus segmentations in the template image [1]. In this way, a correct fitting of the meniscus shapes was achieved, even without initial overlap. The optimization problem was solved using a matrix-free implemented Gauss–Newton method, which uses a multilevel approach to overcome local minima and improve the overall performance. As a result, a dense deformation field that describes the motion for a respective voxel in the template image was obtained. The deformation field was cropped with the menisci masks of the template image.

**Fig. 2:**
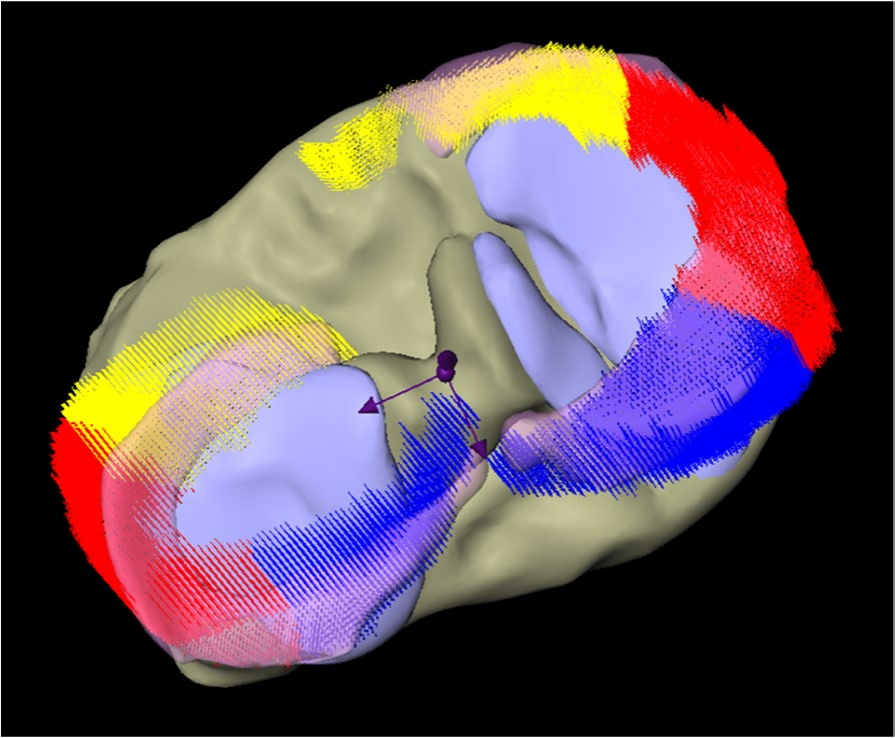
Representation of meniscus movements of different meniscus segments in a coordinate system spanned by mediolateral and anteroposterior axes. * Left: lateral meniscus; right: medial meniscus; yellow: anterior horn; red: intermediate horn; blue: posterior horn. ** Arrows represent mediolateral and anteroposterior axis

The menisci were partitioned equally into 3 parts with respect to the length of the arch. In each of the partitions, the average motion of the voxels from the deformation field was determined. Next, the projection of these motions onto the anterior–posterior axis and the medio-lateral axis, which are automatically obtained from a principal component analysis of the menisci masks, was computed (Fig. 2).

### Statistical analysis

The descriptive statistics are presented in mean values and standard deviations. Differences between the different subgroups were analyzed by using the Wilcoxon signed-rank test. A *p*-value less than 0.05 was considered statistically significant.

Statistical analyses were carried out using IBM SPSS Statistics Version 27.0.0.0.

(IBM Corp., Armonk, New York). The results of the statistical tests were interpreted in an exploratory sense. No adjustment for multiple testing was performed in this exploratory study.

## Results

In the following, the relevant results are summarized. The meniscus movements of both menisci (medial meniscus, MM; lateral meniscus LM) and the respective segments (anterior horn, AH; intermediate horn, IH; posterior horn, PH) are given as the length of the total vector (Avg) and as movement in the mediolateral (ML) and anteroposterior (AP) direction. All movements are given in millimeter (mm).

### Lateral meniscus, internal rotation

**Fig. 3:**
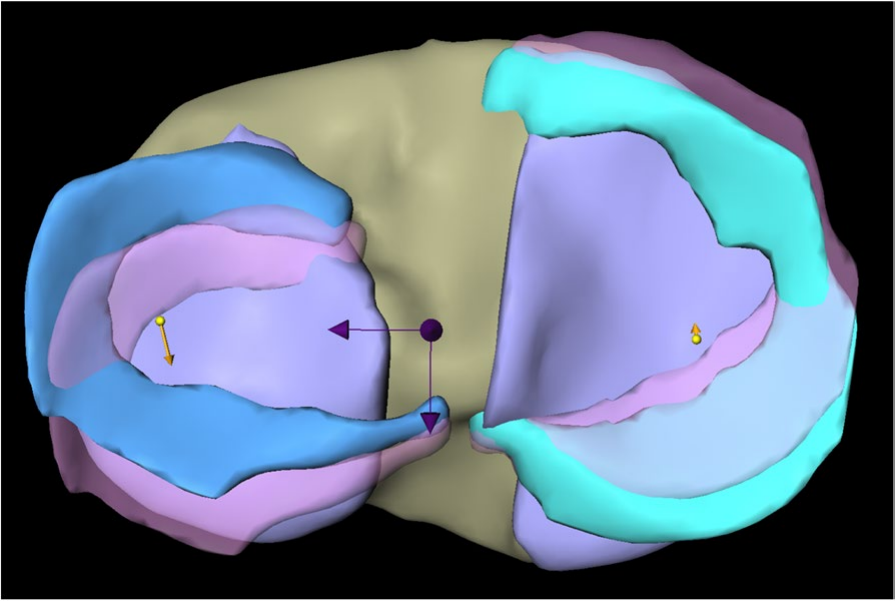
Representation of overall meniscus movements of medial and lateral meniscus in internal rotation. *Left: lateral meniscus; right: medial meniscus; yellow arrows: overall meniscus movements of lateral and medial meniscus. ** Dark arrows represent the mediolateral and anteroposterior axis

The total vector length of the LM motion in internal rotation was 4.55 mm (SD ± 1.19) with an average movement of 1.19 mm (SD ± 0.71) in medial and 4.32 mm (SD ± 1.15) in posterior direction (Fig. 3).

**Fig. 4:**
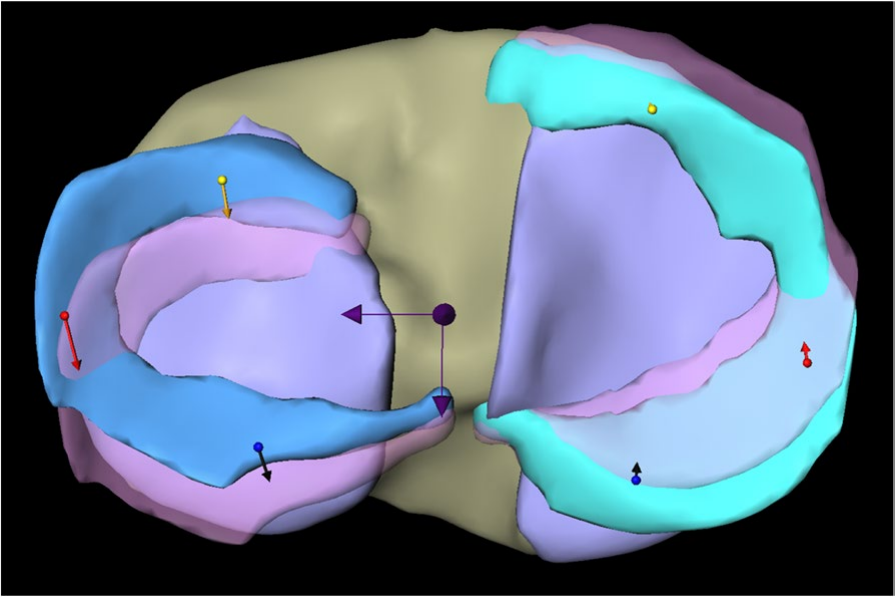
Representation of meniscus movements in different segments of medial and lateral meniscus in internal rotation. *Left: lateral meniscus; right: medial meniscus; yellow arrows: meniscus movements in the anterior horn of lateral and medial meniscus; red arrows: meniscus movements in the intermediate horn of lateral and medial meniscus; blue arrows: meniscus movements in the posterior horn of lateral and medial meniscus. ** Dark arrows represent the mediolateral and anteroposterior axis

The analysis of the different meniscus segments is presented in Table 1 (Fig. 4).

**Table 1:**
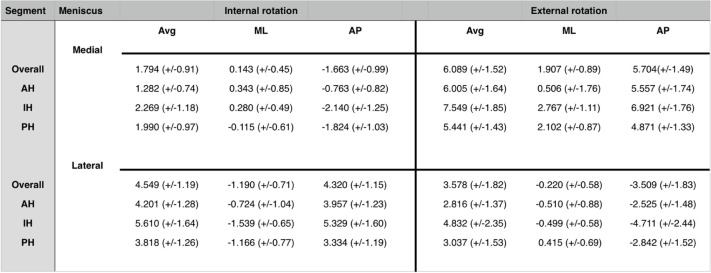
Total meniscus movements (in mm), internal and external rotation

^*^Avg Average, total length of vector, *ML* Meniscus movement in mediolateral direction, *AP* Meniscus movement in anteroposterior direction, *AH* Anterior horn, *IH* Intermediate horn, *PH* Posterior horn, all values are given in mm

^**^A positive value in ML direction corresponds to a movement in lateral direction (negative in medial direction), a positive value in AP direction corresponds to a movement in posterior direction (negative in anterior direction)

^***^values in parenthesis represent standard deviations

### Medial meniscus, internal rotation

The length of the total vector in the analysis of the internal rotational movement of the MM was 1.79 mm (SD ± 0.91) with an average movement of 0.14 mm (SD ± 0.45) in lateral and 1.66 mm (SD ± 0.99) in anterior direction (Fig. 3).

The analysis of the different meniscus segments is presented in Table 1 (Fig. 4).

### Lateral meniscus, external rotation

**Fig. 5:**
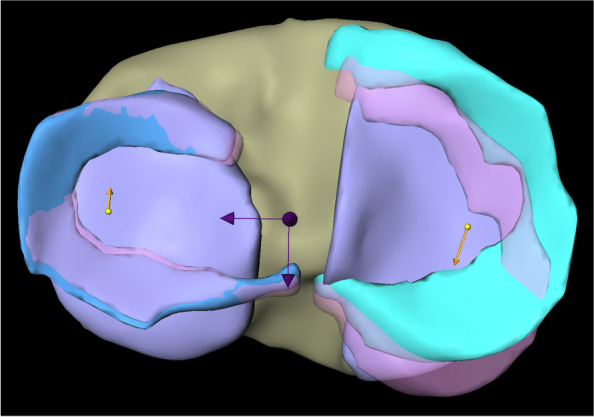
Representation of overall meniscus movements of medial and lateral meniscus in external rotation. * Left: lateral meniscus; right: medial meniscus; yellow arrows: overall meniscus movements of lateral and medial meniscus. ** Dark arrows represent the mediolateral (left arrow) and anteroposterior (back arrow) axis

In the analysis of external rotational movement, the total vector length of the LM motion was 3.58 mm (SD ± 1.82) with an average movement of 0.22 mm (SD ± 0.58) in medial and 3.51 mm (SD ± 1.83) in anterior direction (Fig. 5).

**Fig. 6:**
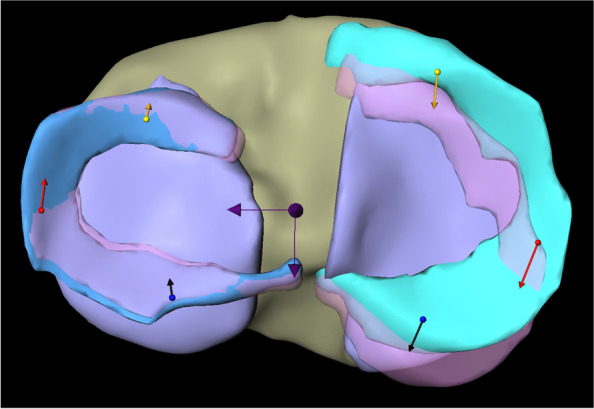
Representation of meniscus movements in different segments of medial and lateral meniscus in external rotation. * Left: lateral meniscus; right: medial meniscus; yellow arrows: meniscus movements in the anterior horn of lateral and medial meniscus; red arrows: meniscus movements in the intermediate horn of lateral and medial meniscus; blue arrows: meniscus movements in the posterior horn of lateral and medial meniscus. ** Dark arrows represent the mediolateral and anteroposterior axis

The analysis of the different segments is presented in Table 1 (Fig. 6).

### Medial meniscus, external rotation

When analyzing external rotational movement, the MM showed a total motion of 6.09 mm (SD ± 1.52) with an average movement of 1.91 mm (SD ± 0.89) in lateral and 5.70 mm (SD ± 1.49) in posterior direction (Fig. 5).

The analysis of the different segments is presented in Table 1 (Fig. 6).

### Medial meniscus, internal vs. external rotation

When investigating differences in meniscus movements of the MM between internal and external rotation using a Wilcoxon signed-rank test, statistically significant differences were found for both the total length of the vector (*p* < 0.05) and the direction of total movement in ML (*p* < 0.05) and AP (*p* < 0.05) direction.

The analysis of the different segments also showed statistically significant differences in meniscus movements between internal and external rotations in almost all the examined parameters. Only the ML movement in the AH did not differ statistically significant (*p* = 0.66) (Table 2).

**Table 2:**
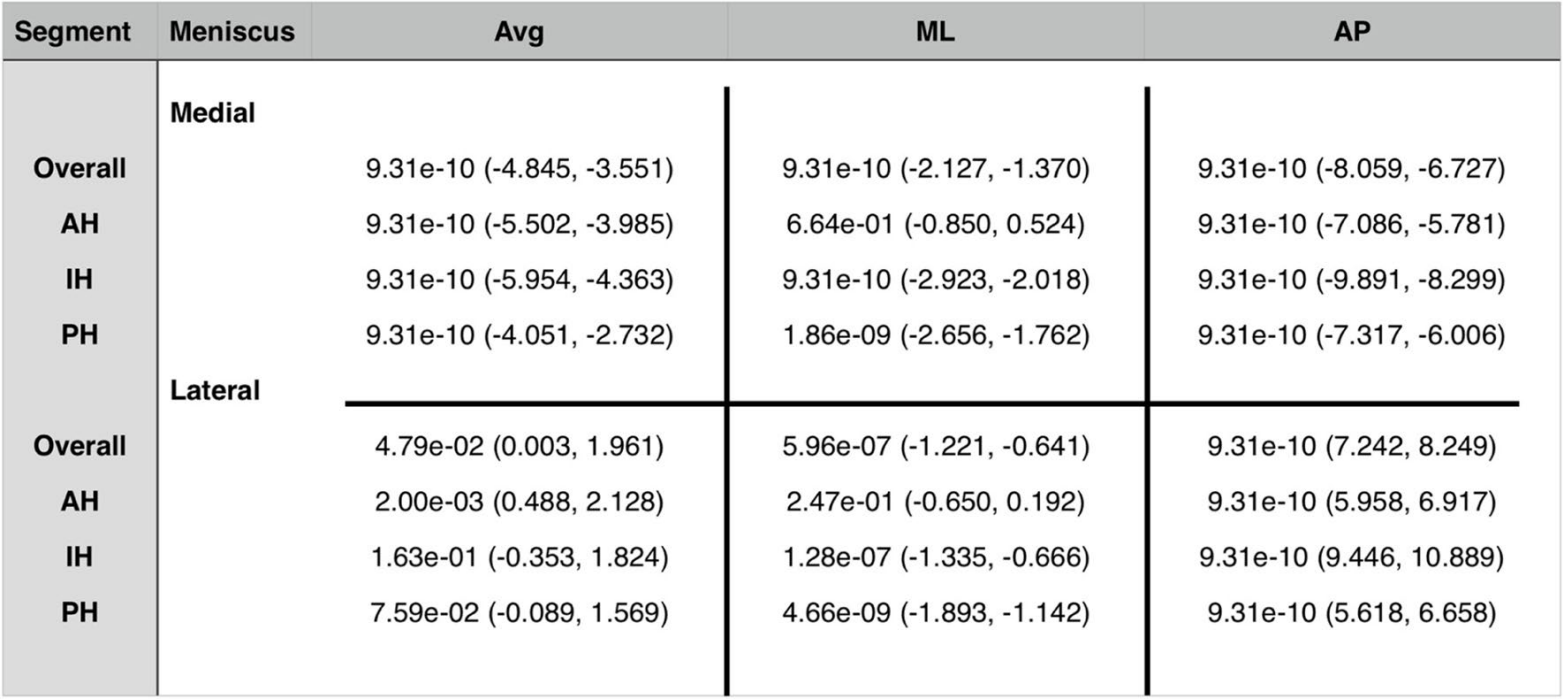
Wilcoxon signed-rank test results of MM and LM movements in internal versus external rotation

^*^Avg Average, total length of vector, *ML* Meniscus movement in mediolateral direction, *AP* Meniscus movement in anteroposterior direction, *AH* Anterior horn, *IH* Intermediate horn, *PH* Posterior horn

^**^Values before parenthesis represent p-values of Wilcoxon signed-rank test; values in parenthesis represent confidence intervals

### Lateral meniscus, internal vs. external rotation

When comparing meniscus movements of the LM between internal and external rotations using a Wilcoxon signed-rank test, statistically significant differences were found in the total length of the vector (*p* < 0.05), as well as in the ML (< 0.05) and AP (*p* < 0.05) direction.

A segment analysis of LM movements in internal versus external rotation showed a statistically significant difference of the total vector length between internal and external rotations in the AH (*p* < 0.05), but not in the IH (*p* = 0.16) or the PH (*p* = 0.08). The direction of meniscus movements showed a statistically significant difference in almost all segments, with only the meniscus movements in the AH in ML direction showing no statistically significant difference (*p* = 0.25) between internal and external rotation (Table 2).

### Internal rotation, medial meniscus vs. lateral meniscus

The analysis of meniscus movements in maximum internal rotation revealed significant differences between MM and LM, both in terms of total vector length (*p* < 0.05) and in relation to the meniscus movements in the ML (*p* < 0.05) and AP (*p* < 0.05) directions.

When differentiating between segments, statistically significant differences between MM and LM in internal rotation could be demonstrated in all segments, both in terms of vector length and direction (mediolateral and anteroposterior) (Table 3).

**Table 3:**
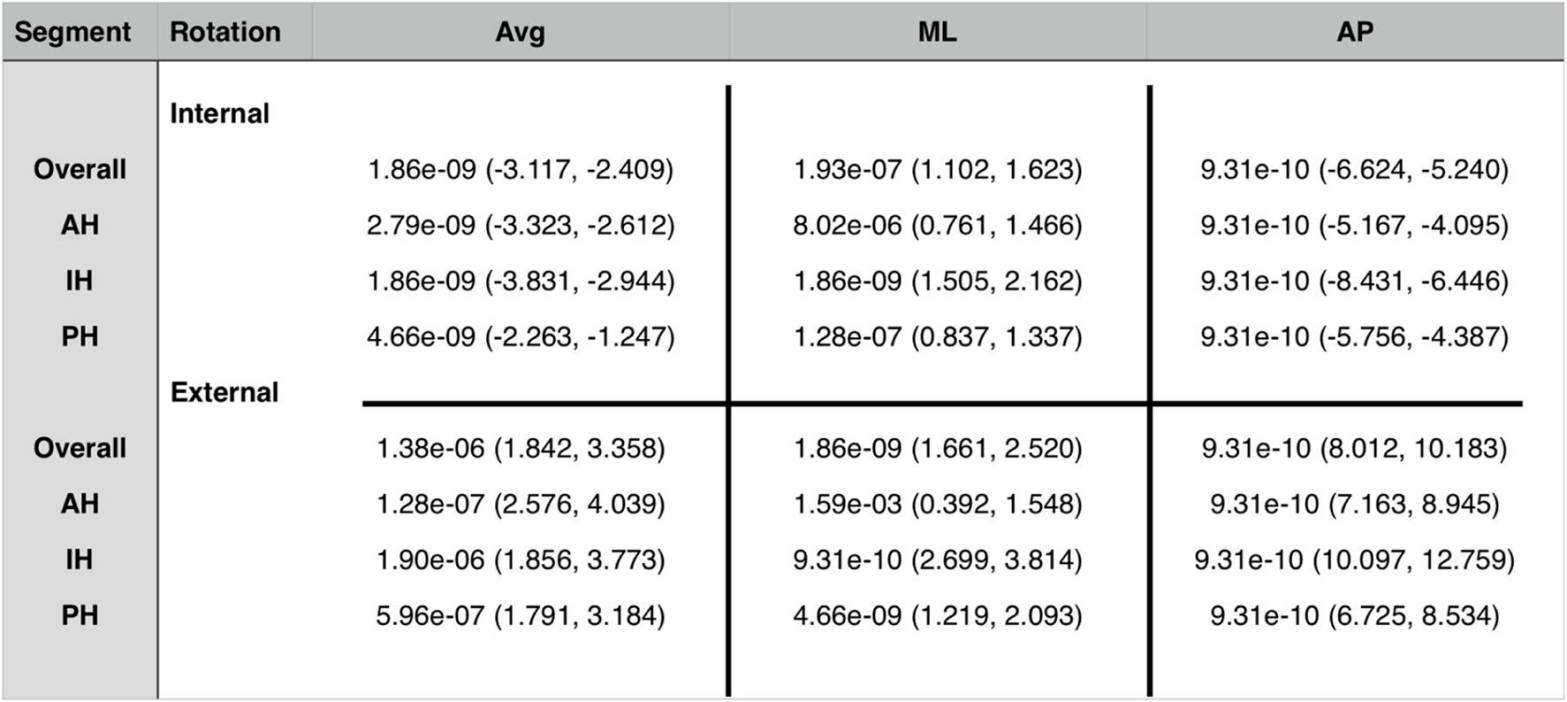
Wilcoxon signed-rank test results of MM versus LM movements in internal and external rotation

^*^Avg Average, total length of vector, *ML* Meniscus movement in mediolateral direction, *AP* Meniscus movement in anteroposterior direction, *AH* Anterior horn, *IH* Intermediate horn, *PH* Posterior horn

^**^Values before parenthesis represent *p*-values of Wilcoxon signed-rank test; values in parenthesis represent confidence intervals

### External rotation, medial meniscus vs. lateral meniscus

The comparison between MM and LM movements in maximum external rotation also showed statistically significant differences in vector length (*p* < 0.05), ML (*p* < 0.05) and AP direction (*p* < 0.05).

A segment analysis of MM versus LM movements in external rotation also showed statistically significant differences in all segments, both in terms of vector length and direction (mediolateral and anteroposterior) (Table 3).

## Discussion

The most important findings of this study are that:

- 1. Meniscus movements of MM and LM in internal and external rotations differ significantly in extent and direction; MM and LM move in opposite directions during internal and external rotation.
- 2. In internal rotation, most meniscus movements were found in the IHLM. In external rotation, the IHMM showed the greatest mobility.
- 3. A segment analysis of internal and external rotation showed fewer differences in the movements of LM than MM.

In the present study, the MM showed an average movement of 1.79 mm in anterior-lateral direction in internal rotation and 6.01 mm posterior-lateral in external rotation, whereas the LM moved an average of 4.55 mm posterior-medial in internal and 3.58 mm anterior-medial in external rotation. Overall, these movements, both in MM and LM, showed a greater extent in AP direction than ML, where there was a clear difference in the extent of the translational (AP) movement of MM. The posterior movement of the entire MM in external rotation was 5.70 mm, whereas the anterior movement in internal rotation was only 1.66 mm. Previous in-vivo studies support these findings, which show that radial movement of the menisci is less pronounced than translational movement for both the MM and the LM [2, 11].

In the study collective presented here, external rotation caused increased meniscal movement of MM structures when compared with the LM. In external rotation, the MM moves in a posterior-lateral and the LM in an anterior-medial direction. In internal rotation, the LM showed a higher overall movement (which was directed posteriorly and medially), while the MM moved in an anterior-lateral direction.

In internal rotation, the most meniscus movement was found in the IHLM. In external rotation, the IHMM showed the greatest mobility. Here, with a vector length of 7.55 mm, the greatest average mobility of the examined segments was seen.

Although multiple researchers have investigated the dynamic characteristics of the menisci during knee joint motion, there is little evidence of meniscal movements in internal and external rotation [2, 10, 11, 25]. Current literature on the exact movement of different meniscus segments during rotation of the knee joint rotation is not available. Nevertheless, a review of the literature reveals some points which are worth discussing.

Boxheimer et al. investigated 22 knees from 22 asymptomatic volunteers with a 0.5-T open-configuration MR system. Sagittal and coronal images were obtained with the knee in different positions. The position of the menisci from the outer inferior edge of the meniscus to the outermost edge of the articular cartilage of the tibial plateau was measured, and meniscal movement was then calculated [2]. The authors postulated that meniscal movement is most prominent in the AHMM, which differs from the findings of this study, where most meniscus movements were found in the IHMM (external rotation), followed by the IHLM (internal rotation). The extent of total meniscus movements depends, naturally, on the size of the defined meniscus section. A standardized differentiation in the respective study setting between AH, IH and PH, as provided in this study, is therefore of great importance. Together with the different, non-validated measuring techniques and references, this may explain the different findings. The exact determination of movements in the different meniscus segments depending on torsional movements could be of particular importance in the detection of specific injuries in the future. A relevant clinical reference is the detection of so-called “hidden lesions” in the area of meniscocapsular attachment. Sonnery-Cottet et al. assumed that these meniscal ramp lesions occur much more frequently than it was previously thought, particularly in ACL-injured knees. The authors interpreted the rate of missed diagnoses as a result of unfamiliarity with this injury pattern within the orthopedic community, and therefore the difficulty in diagnosis [20]. Similar results were also published by Jacquet et al., for instabilities of the PHLM in ACL-deficient knee joints. The authors postulated an arthroscopically detected instability of the PHLM in about one third of the patients with ACL instability [8].

A validated method for MRI in-vivo investigation of potentially pathological movements in the PHMM and PHLM could close this diagnostic gap. Indeed, a meniscal tear in this area might only be considered relevant when the result is a pathological movement pattern. But before such a reproducible detection of pathological meniscus movements can be established, the physiological meniscus movements must first be described in a validated setting. The above-mentioned parameters for this experimental observation where therefore chosen to describe physiological meniscus movements with ensuring the best possible reproducibility of the test setup and not to simulate movements with increased susceptibility to injury of the meniscus tissue.

Jacquet et al. acknowledged the diagnostic gap in detection of lateral meniscus posterior horn instability with the “aspiration test”, which can demonstrate instability intraoperatively. The setting used in this study offers the possibility of supplementing the gold standard of arthroscopic evaluation with MRI [7]. Studies which investigate meniscal motion in-vivo used open MRI, because the limited space of a closed bore cannot provide adequate controlled movement [2, 25]. The occurrence of movement artifacts, which regularly occurs in dynamic MRI examinations, was reduced with prospective motion correction [12], thus, ensuring high image quality, essential for evaluation and semi-automated segmentation. Measurements were performed with a single-slice method in most of the previous studies to determine the meniscal position in MRI [2, 4, 27]. Certain anatomical landmarks were identified to select the particular slice, and parallel lines were drawn at the outer edge of the cartilage and the peripheral rim of the meniscus. Depending on execution and slice selection, this method could under- or overestimate meniscal extrusion [9]. Furthermore, evaluating only one slice and one edge of a three-dimensional deformable structure can impair results [23]. Wirth et al. introduced a 3D method to determine the meniscal position in relation to its contact area and morphology. It allowed for the measurement of meniscal extrusion globally in all areas of the knee joint [28]. To exhibit motion of the meniscus, Yao et al. proposed a 3D matrix volume with calculated centroids, where a change of centroids was able to detect and quantify the meniscal motion [29].

The 3D voxel vector-based approach used in this study allows for the determination of motion in all of the meniscus, with direction and a range of the movement. Superior 3D-based methods can determine the position and motion of the meniscus accurately, improve understanding of meniscal physiological structure and function, and accurately detect pathologies.

One limitation of this study is that femoral soft tissue (such as muscle and fat) which makes adequate fixation difficult could have delayed or impaired movement of the knee joint in the desired direction. Additionally, involuntary evasive movements of the test subject with passive movement of the leg may have led to inaccuracies. However, neither should interfere significantly with the relative change in dynamics between tibia and femur. Finally, with a cohort of 31 volunteers no adapted statistical analyses with covariates like height, BMI or age was performed. A larger study cohort would allow for future studies with these co-factors.

## Conclusions

Movements of MM and LM in internal and external rotation differ significantly in extent and direction, whereas MM and LM movements in opposite directions during internal and external rotation can be observed. In internal rotation, most meniscus movements were found in the IHLM. In external rotation, the IHMM showed the greatest level of mobility. A segment analysis of internal vs. external rotation showed fewer differences of LM movements than MM.

## Data Availability

All relevant data are provided within the manuscript. The datasets used and/or analyzed during the current study are available from the corresponding author on reasonable request.

## Abbreviations

MM: Medial meniscus
LM: Lateral meniscus
AH: Anterior horn
IH: Intermediate horn
PH: Posterior horn
AP: Anteroposterior
ML: Mediolateral
MRI: Magnetic resonance imaging
PMC: Prospective motion correction
BMI: Body mass index
MPT: Moiré phase tracking
SSD: Sum-of-squared-differences

## References

[1] Audette MA, Ferrie FP, Peters TM (2000). An algorithmic overview of surface registration techniques for medical imaging. Med Image Anal.

[2] Boxheimer L, Lutz AM, Treiber K, Goepfert K, Crook DW, Marincek B (2004). MR imaging of the knee: position related changes of the menisci in asymptomatic volunteers. Invest Radiol.

[3] Bylski-Austrow DI, Ciarelli MJ, Kayner DC, Matthews LS, Goldstein SA (1994). Displacements of the menisci under joint load: an in vitro study in human knees. J Biomech.

[4] Crema MD, Roemer FW, Felson DT, Englund M, Wang K, Jarraya M (2012). Factors associated with meniscal extrusion in knees with or at risk for osteoarthritis: the Multicenter Osteoarthritis study. Radiology.

[5] Fischer B, Modersitzki J (2003). Curvature Based Image Registration. J Math Imaging Vis.

[6] Fox AJ, Wanivenhaus F, Burge AJ, Warren RF, Rodeo SA (2015). The human meniscus: a review of anatomy, function, injury, and advances in treatment. Clin Anat.

[7] Jacquet C, Magosch A, Mouton C, Seil R (2021). The aspiration test: an arthroscopic sign of lateral meniscus posterior horn instability. J Exp Orthop.

[8] Jacquet C, Mouton C, Magosch A, Komnos GA, Menetrey J, Ollivier M (2022). The aspiration test reveals an instability of the posterior horn of the lateral meniscus in almost one-third of ACL-injured patients. Knee Surg Sports Traumatol Arthrosc.

[9] Jones LD, Mellon SJ, Kruger N, Monk AP, Price AJ, Beard DJ (2018). Medial meniscal extrusion: a validation study comparing different methods of assessment. Knee Surg Sports Traumatol Arthrosc.

[10] Kawahara Y, Uetani M, Fuchi K, Eguchi H, Hashmi R, Hayashi K (2001). MR assessment of meniscal movement during knee flexion: correlation with the severity of cartilage abnormality in the femorotibial joint. J Comput Assist Tomogr.

[11] Kawahara Y, Uetani M, Fuchi K, Eguchi H, Hayashi K (1999). MR assessment of movement and morphologic change in the menisci during knee flexion. Acta Radiol.

[12] Lange T, Maclaren J, Herbst M, Lovell-Smith C, Izadpanah K, Zaitsev M (2014). Knee cartilage MRI with in situ mechanical loading using prospective motion correction. Magn Reson Med.

[13] Lange T, Taghizadeh E, Knowles BR, Sudkamp NP, Zaitsev M, Meine H (2019). Quantification of patellofemoral cartilage deformation and contact area changes in response to static loading via high-resolution MRI with prospective motion correction. J Magn Reson Imaging.

[14] Maclaren J, Armstrong BS, Barrows RT, Danishad KA, Ernst T, Foster CL (2012). Measurement and correction of microscopic head motion during magnetic resonance imaging of the brain. PLoS One.

[15] Masouros SD, McDermott ID, Amis AA, Bull AM (2008). Biomechanics of the meniscus-meniscal ligament construct of the knee. Knee Surg Sports Traumatol Arthrosc.

[16] Mastrokalos DS, Papagelopoulos PJ, Mavrogenis AF, Hantes ME, Karachalios TS, Paessler HH (2005). Changes of meniscal interhorn distances: an in vivo magnetic resonance imaging study. Knee.

[17] Patel VV, Hall K, Ries M, Lotz J, Ozhinsky E, Lindsey C (2004). A three-dimensional MRI analysis of knee kinematics. J Orthop Res.

[18] Poulsen E, Goncalves GH, Bricca A, Roos EM, Thorlund JB, Juhl CB (2019). Knee osteoarthritis risk is increased 4–6 fold after knee injury - a systematic review and meta-analysis. Br J Sports Med.

[19] Shefelbine SJ, Ma CB, Lee KY, Schrumpf MA, Patel P, Safran MR (2006). MRI analysis of in vivo meniscal and tibiofemoral kinematics in ACL-deficient and normal knees. J Orthop Res.

[20] Sonnery-Cottet B, Serra Cruz R, Vieira TD, Goes RA, Saithna A (2020). Ramp Lesions: An Unrecognized Posteromedial Instability?. Clin Sports Med.

[21] Stehling C, Souza RB, Hellio Le Graverand MP, Wyman BT, Li X, Majumdar S (2012). Loading of the knee during 3.0T MRI is associated with significantly increased medial meniscus extrusion in mild and moderate osteoarthritis. Eur J Radiol.

[22] Thompson WO, Thaete FL, Fu FH, Dye SF (1991). Tibial meniscal dynamics using three-dimensional reconstruction of magnetic resonance images. Am J Sports Med.

[23] Tibesku CO, Mastrokalos DS, Jagodzinski M, Passler HH (2004). MRI evaluation of meniscal movement and deformation in vivo under load bearing condition. Sportverletz Sportschaden.

[24] Tienen TG, Buma P, Scholten JG, van Kampen A, Veth RP, Verdonschot N (2005). Displacement of the medial meniscus within the passive motion characteristics of the human knee joint: an RSA study in human cadaver knees. Knee Surg Sports Traumatol Arthrosc.

[25] Vedi V, Williams A, Tennant SJ, Spouse E, Hunt DM, Gedroyc WM (1999). Meniscal movement. An in-vivo study using dynamic MRI. J Bone Joint Surg Br.

[26] Walker PS, Arno S, Bell C, Salvadore G, Borukhov I, Oh C (2015). Function of the medial meniscus in force transmission and stability. J Biomech.

[27] Winkler PW, Wierer G, Csapo R, Hepperger C, Heinzle B, Imhoff AB (2020). Quantitative Evaluation of Dynamic Lateral Meniscal Extrusion After Radial Tear Repair. Orthop J Sports Med.

[28] Wirth W, Frobell RB, Souza RB, Li X, Wyman BT, Le Graverand MP (2010). A three-dimensional quantitative method to measure meniscus shape, position, and signal intensity using MR images: a pilot study and preliminary results in knee osteoarthritis. Magn Reson Med.

[29] Yao J, Lancianese SL, Hovinga KR, Lee J, Lerner AL (2008). Magnetic resonance image analysis of meniscal translation and tibio-menisco-femoral contact in deep knee flexion. J Orthop Res.

[30] Zaitsev M, Dold C, Sakas G, Hennig J, Speck O (2006). Magnetic resonance imaging of freely moving objects: prospective real-time motion correction using an external optical motion tracking system. Neuroimage.

